# Structural Priming Treatment in Aphasia: The Role of Lexical and Abstract Syntactic Representations

**DOI:** 10.1044/2025_AJSLP-25-00095

**Published:** 2025-10-22

**Authors:** Willem S. van Boxtel, Katelin M. Rainey, Victor Ferreira, Nadine Martin, Emily Bauman, Jiyeon Lee

**Affiliations:** a Department of Speech, Language, and Hearing Sciences, Purdue University, West Lafayette, IN; b Department of Communication Sciences & Disorders, Louisiana State University, Baton Rouge; c Department of Psychology, University of California San Diego; d College of Public Health, Temple University, Philadelphia, PA

## Abstract

**Purpose:**

Sentence production is impaired in many persons with aphasia (PWA). However, few effective treatments for sentence production exist. Recent research has advanced *structural priming* as a promising treatment for aphasia, but the underlying mechanisms of priming remain unclear. This study examined contributions of abstract syntactic and lexically boosted priming to sentence production improvements in PWA and underlying memory mechanisms.

**Method:**

Twenty-four PWA and 16 age-matched controls completed baseline testing, three to six sessions of sentence production priming training, and 1-day and 1-week posttesting. Trained structures were passives and double-object datives. Participants were trained with same-verb and different-verb priming to assess lexical boost and abstract syntactic priming effects on treatment outcomes. The serial reaction time, fragmented picture, and picture pointing span tests were administered to assess contributions of implicit and explicit memory in predicting treatment gains.

**Results:**

PWA and controls showed lasting improvements to both trained and untrained sentences following training. Critically, controls improved more strongly following same-verb priming, while PWA showed stronger gains following different-verb priming. High implicit memory scores facilitated greater treatment effects in both PWA and controls. Only controls showed positive effects of explicit memory.

**Conclusions:**

These results support structural priming as an effective sentence production treatment for PWA, especially when verbs are not matched between primes and target. We suggest lexical differentiation supports priming in PWA by allowing more efficient access and learning of abstract syntactic representations, which appears crucial to successful sentence production.

Aphasia is a multifaceted language disorder that affects various aspects of communication, with significant negative impact on the quality of life in many affected individuals (Dalemans et al., 2010; Hilari et al., 2012). Difficulty producing sentences is commonly seen in persons with aphasia (PWA), affecting their use of simple and complex sentences (e.g., Cho-Reyes & Thompson, 2012). While various theoretical accounts of these impairments are available, one dominant view is that the processes of mapping or encoding message elements onto sentence structures are affected in PWA (Bastiaanse & van Zonneveld, 2004; Lee et al., 2015; Schwartz et al., 1994; Thompson et al., 2015). During these message-syntax mapping processes, speakers assign thematic roles (e.g., agent, theme, goal) to lexical items, encoding “who is doing what to whom” and assemble them into a correct syntactic structure following grammatical constraints of the language (e.g., Bock & Levelt, 1994; Levelt, 1989).

PWA with such mapping deficits may show various production difficulties. Studies suggest that PWA show impaired ability to encode complete verb argument structures in sentences, yielding omissions of obligatory arguments (e.g., “The man fixing” for “The man is fixing the car”) or incorrect production of argument structure (e.g., “The prince gave to Cinderella the shoes”; Berndt, 2015; Kim & Thompson, 2004; Marshall et al., 1998; Thompson, 2003). PWA may also make role reversal errors, particularly when sentences are semantically reversible, indicating impaired thematic role assignment (e.g., “The girl is chasing the boy” for “The boy is chasing the girl”; Lee et al., 2015, 2023; Lee, Man, et al., 2019; Marshall, 1995; Schwartz et al., 1994). Difficulty producing sentences with noncanonical word orders in PWA is particularly well known (Caplan & Hanna, 1998; Cho-Reyes & Thompson, 2012; Cho-Reyes et al., 2016; Faroqi-Shah & Thompson, 2003; Havik & Bastiaanse, 2004; Kirshner & Jacobs, 1996; Knibb et al., 2009; Mack et al., 2017; Miceli et al., 1989; Schwartz et al., 1994). For example, in the English language, it has been consistently shown that PWA have difficulty with passive sentences because the theme noun is produced in the subject position (“the man is bitten by the dog”) in passives, thus requiring greater syntactic processing, compared to active sentences (“The dog is biting the man”), which follow the canonical word order. Increased difficulty associated with noncanonical mapping in PWA extends to irreversible sentences, such as double-object (DO) datives (“The man gives the dog a treat”), compared to the canonical prepositional object (PO) structure (“The man gives a treat to the dog”; e.g., Cho-Reyes et al., 2016; Man et al., 2019).

Effective treatments for sentence production deficits in aphasia are nevertheless lacking. The few existing treatments generally follow highly explicit, drill-based teaching of grammatical rules. For instance, in linguistically based treatments such as the Treatment of Underlying Forms program (Thompson & Shapiro, 2005; Thompson et al., 2003) and Mapping Therapy (Rochon et al., 2005; Schwartz et al., 1994), a clinician explicitly trains PWA to identify the syntactic relations of different sentence constituents (e.g., verbs and nouns) and to formulate specific target sentences using those grammatical rules. This explicit learning of grammatical rules may not be suitable for some PWA, especially when their verbal short-term or working memory is compromised (Caplan et al., 2013; Martin et al., 2018). Recent research has advanced *structural priming*, a phenomenon in which exposure to a “prime” sentence with a specific grammatical structure facilitates subsequent production of “target” sentences with that structure, as a promising strategy that facilitates implicit grammatical learning for aphasia (e.g., Lee, Van Boxtel, et al., 2025; Rainey et al., 2024; see Lee, 2024, for a review). Single-subject–based treatment programs using structural priming, for example, have yielded treatment gains that can last up to 2 months (Lee & Man, 2017; Rainey et al., 2024). In a more recent study, Lee, Van Boxtel, et al. (2025) found that a group of 16 PWA showed improved production of trained and untrained passive and dative sentences at 1-week posttesting, after a brief training (three sessions) of implicit structural priming.

While increasing evidence suggests structural priming as a promising treatment for PWA, its positive effects need to be replicated in a larger number of studies, and its essential components that support robust treatment gains have yet to be specified. For example, in healthy adults, priming-induced facilitation is greatly amplified by introducing lexical head (e.g., verb) overlap between prime and target sentences, an effect known as the *lexical boost* (Hartsuiker et al., 2008; Tooley, 2020). Findings from PWA are less uniform. Some studies have found intact lexical boost effects in PWA (Lee et al., 2023; Yan et al., 2018; see also Zhang & Lee, 2024), while others have failed to discover significant lexically boosted priming in PWA (e.g., Lee, Man, et al., 2019; Man et al., 2019; Van Boxtel et al., 2023). This study sought to further validate whether structural priming is an effective treatment for sentence-level deficits in aphasia and to examine if and how lexical–syntactic interactions modulate the strength and longevity of treatment effects.

## Structural Priming as a Treatment Component

In cognitive science, *priming* refers to changes in processing behavior pertaining to specific cues or items, resulting from prior exposure to that cue or similar cues (Schacter & Buckner, 1998). Priming occurs at many different levels of processing, including *semantic priming*, in which exposure to specific semantic items facilitates processing of semantically similar items later on (Hutchison et al., 2013); *phonological priming*, in which prior experience with phonological items facilitates processing of the same phonological item subsequently (Wilson et al., 2011); and *structural* or *syntactic priming*, which is the focus of the current work. When speakers hear or read an utterance with a specific grammatical structure that maps a message onto a certain word order (e.g., a passive sentence), subsequent processing of that structure is facilitated, and speakers are more likely to use that structure in later utterances. This effect is highly robust and has been attested in sentence production (Bock, 1986; Mahowald et al., 2016; Pickering & Ferreira, 2008), sentence comprehension (Tooley et al., 2014; Tooley & Traxler, 2010; Traxler & Tooley, 2008), across production and comprehension modalities (Keen & Lee, 2024; Litcofsky & Hell, 2019), and even across languages (Hartsuiker et al., 2004; Loebell & Bock, 2003).

Priming effects have been elicited in a wide variety of age groups, from young children (Huttenlocher et al., 2004; Messenger, 2021) to college-aged groups (Mahowald et al., 2016) and older adults (Lee et al., 2024; Van Boxtel & Lawyer, 2023a, 2023b). In addition, patients with amnesia exhibit structural priming effects (Ferreira et al., 2008; Heyselaar et al., 2017), and crucially, structural priming has been shown to be intact in PWA (Hartsuiker & Kolk, 1998; Lee, Man, et al., 2019; Saffran & Martin, 1997). Priming can be operationalized in a variety of experimental and training paradigms. Neurologically healthy participants as well as PWA show increased likelihoods of producing primed compared to unprimed structures when describing pictures (e.g., Van Boxtel et al., 2023), faster reading times when reading sentences (e.g., Man, 2023; Tooley & Traxler, 2010), and preferences for primed interpretations of ambiguous syntax (e.g., Lee, Hosokawa, et al., 2019). Priming can be induced through sentence repetition (e.g., Yan et al., 2018), a sentence–picture matching task (e.g., Lee, Hosokawa, et al., 2019; Van Boxtel et al., 2023), or a dialogue-like interactive task (e.g., Man et al., 2019; Lee et al., 2023). In short, priming is ubiquitous and can be recorded using a wide variety of measures.

Two critical features of structural priming highlight its potential applicability to sentence production treatments. First, structural priming happens implicitly; that is, speakers are generally unaware that they are repeating primed syntactic structures in their own production (K. Bock & Griffin, 2000; Chang et al., 2006, 2012). Priming requires no explicit instructions on which sentence structures to use, or any explicit recall of prime sentences (Bock et al., 1992; Branigan & Messenger, 2016; Ferreira et al., 2008; Reitter & Moore, 2014). The implicit nature of priming could be beneficial for PWA whose explicit metacognitive skills to follow multistep rules of therapy are compromised (see, e.g., Schuchard et al., 2017; Schuchard & Thompson, 2014, for evidence of preserved implicit learning in PWA). Second, a large number of studies show that structural priming can be a *cumulative* and *long-lasting* process: While priming effects are robust after only one exposure to a prime sentence (see Mahowald et al., 2016, for a review), additional exposures reinforce priming effects (Fine & Jaeger, 2016; Kaschak et al., 2011, 2014). Such cumulatively larger priming effects not only support the reliance of priming on implicit learning (e.g., Heyselaar et al., 2021) but may also make priming even more applicable to sentence production treatments for aphasia. In addition, priming effects are observable even if significant time (e.g., 1 month; see Bernolet et al., 2016; Messenger, 2021) or intervening filler material (e.g., four unrelated sentences; see Scheepers, 2003; Van Boxtel & Lawyer, 2023b) intercedes between prime and target in healthy speakers. These long-term effects reported in previous studies with neurotypical populations provide a promising empirical basis for developing priming-based treatment programs for PWA.

Subsequently, several recent studies from our group have begun to elucidate which components of structural priming can be manipulated to increase its efficacy as a treatment component (Lee, Man, et al., 2025; Lee, Van Boxtel, et al., 2025; Rainey et al., 2024). These studies yielded promising results: Overall, structural priming treatment caused performance on independent sentence production measures to increase significantly from a pretreatment baseline session to posttesting sessions. In the Lee, Van Boxtel, et al. (2025) study, treatment sessions consisted of participants listening to prime sentences before reading those sentences out loud. After three treatment sessions, both PWA and age-matched controls showed higher accuracy on treated and untreated sentences and a greater likelihood to produce noncanonical sentence structures (passives and DOs) on independent sentence production measures.

It is still unknown what the optimal components of a structural priming treatment are. Lee, Van Boxtel, et al. (2025) established that increasing the number of opportunities to activate the target structure is essential to larger treatment gains in PWA. Their PWA showed greater sentence production improvements when they received single-structure primes, compared to when they received the priming treatment condition in which the target structure was alternated with its nontarget alternative (e.g., some passive and some active prime sentences). Nevertheless, there are many more possible parameters that can be systematically varied and tested in structural priming treatments. One of the most consistently observed additional sources of facilitation in the structural priming literature is lexical amplification (Mahowald et al., 2016). When prime and target sentences share a lexical head (e.g., verb), the magnitude of priming effects is amplified greatly (Hartsuiker et al., 2008; Tooley, 2020; Traxler et al., 2014). This effect, known as the lexical boost, is thought to rely on the spreading activation from syntactic cues to lexical information: When sentences are read, not only is the syntactic structure of that sentence activated, but lexical information is tied to syntactic structure and activated concurrently (Pickering & Branigan, 1998).

The lexical boost is generally thought to be much less long lasting than abstract priming (which can persist across several weeks and multiple intervening sentences; see Bock & Griffin, 2000; Savage et al., 2006). A seminal study by Hartsuiker et al. (2008) suggested that structural priming persists across as many as eight intervening fillers, but the lexical boost begins to decay after just two such intervening sentences. This study was followed by several others (e.g., Tooley, 2020; Traxler et al., 2014), which appeared to confirm the short-lived effects of the lexical boost. This rapid decay might be exacerbated in PWA, whose explicit memory skills are generally reduced and whose access to linguistic representations may be impaired (Martin et al., 2018; Schwartz et al., 1994). However, recent studies employing fine-grained methods such as electroencephalography/event-related potential and careful statistical controls have nuanced the short-lived view of the lexical boost somewhat, suggesting that effects of lexical overlap are, in fact, observable after several fillers and making the case for a unified implicit learning mechanism underlying both abstract priming and the lexical boost (Heyselaar et al., 2021; Van Boxtel & Lawyer, 2023b).

In aphasia, it is unclear whether the lexical boost exists, let alone whether it might amplify cumulative priming-induced sentence production gains. A picture description task including PWA and age-matched controls reported in Yan et al. (2018) found reliable lexical boost effects in both groups, although the magnitude of the lexical boost was somewhat smaller in PWA than controls (see also Lee et al., 2023). Conversely, several later studies found no lexical boost in PWA: Both Man et al. (2019) and Van Boxtel et al. (2023) found no evidence for lexically boosted priming in PWA, despite reporting large lexical boost effects in healthy controls (see further Lee, Man, et al., 2019, who reported no lexical boost effects in either group). These findings cast doubt on whether lexical overlap can amplify existing structural priming effects in PWA; establishing whether the lexical boost is effective will be essential to specifying the optimal treatment components in future priming-based sentence production treatment protocols.

Another focus of the current study is to investigate if and how individual differences in explicit and implicit memory contribute to priming-induced treatment gains. While it is generally agreed that structural priming effects reflect a form of language learning, less is known about the underlying memory processes that support priming-induced learning. Most existing studies assume implicit memory processes underpin abstract priming (e.g., Bock & Griffin, 2000; Chang et al., 2006, 2012) and a different, explicit memory mechanism subserves the lexical boost (e.g., Hartsuiker et al., 2008; Traxler et al., 2014). Evidence for this notion mainly includes the finding that the lexical boost tends to be larger but is far less long-lived than abstract priming, decaying after only a small number of intervening filler sentences (Hartsuiker et al., 2008). However, recent evidence has contradicted this notion (e.g., Van Boxtel & Lawyer, 2023b), leading some researchers to suggest an implicit basis for all aspects of structural priming (see Heyselaar et al., 2021). It therefore remains to be seen to what extent different memory processes support strength and longevity of priming effects in PWA and controls. This study therefore included measures of explicit and implicit memory and associated these with priming effects.

## The Present Study

In this study, we aimed to (a) further validate whether structural priming is an effective treatment for sentence production deficits in aphasia and (b) examine the extent to which different priming conditions (same verb vs. different verb) modulate treatment gains. While structural priming is a highly promising paradigm for sentence production treatments in aphasia, specific mechanisms underlying priming and what components of priming would make treatment optimal are yet to be specified. If lexically boosted priming (same-verb priming) leads to greater treatment gains than abstract (different-verb) structural priming, this would make the case for the lexical boost to be included in future sentence production treatments for aphasia based on priming. Thus, this study comprised a Phase 1 pretrial clinical intervention study (Rogers, 2009) designed to identify optimal treatment components in a structural priming sentence production treatment for aphasia. To this end, we designed a baseline–training–posttesting paradigm similar to that used in Lee, Van Boxtel, et al. (2025), targeting improved production of passive and DO dative sentences.

At baseline, PWA and controls completed sentence production and completion probe tasks. Then, the participants completed either three or six sessions of oral reading structural priming training where they heard and read two prime sentences before completing a target sentence (see the Structural Priming Treatment Task section for more details). The hypothesized mechanism of action was that after reading prime sentences, the prime's syntactic structure (e.g., passive) would be activated and become more accessible for future use, and this accumulative priming would create lasting changes in the sentence production systems of the participants (Bock & Griffin, 2000; Chang et al., 2006, 2012). Baseline probe tasks were then repeated 1 day and 1 week after treatment ceased. Participants also completed implicit and explicit memory tests at baseline to test how individuals' memory scores predict training gains.

Importantly, participants were treated both with same-verb training, in which the prime and target sentences shared a main verb, and different-verb training, in which all verbs were different between primes and targets. The order of the training conditions and target structures counterbalanced across participants within each group (but see the Structural Priming Treatment Task section for some exceptions). Our primary outcome measures were improvements in sentence production and completion probes at 1-day and 1-week posttesting compared to baseline. Additionally, we included untrained target stimuli to assess generalization effects. Lastly, as our secondary measure, we included daily probes, which were administered at the end of each priming training session, to assess gradual improvements on sentence production during the treatment phase.

The following questions were investigated. First, we asked whether PWA and controls show long-term sentence production improvements at 1-day and 1-week posttesting after structural priming treatment. Following Lee, Van Boxtel, et al. (2025), we hypothesized that PWA and controls will show improvements from baseline to 1-day posttesting and maintenance of treatment gains from 1-day to 1-week posttesting. Second, we asked whether verb overlap between prime and target amplifies treatment gains using same-verb and different-verb training conditions. It was hypothesized that controls will show increased priming treatment effects in the same-verb condition, but following unclear past findings about the lexical boost in aphasia, no specific hypotheses about the PWA group were made. Third, we tested generalization of treatment gains from items seen during priming training to untrained items to investigate whether structural priming treatment leads to generalized improvements to sentence production. We hypothesized that full generalization will take place from trained to untrained items, following Lee, Van Boxtel, et al. (2025). Finally, we asked whether individual differences in implicit and explicit memory abilities affected the strength of structural priming treatment gains in PWA and controls and hypothesized that implicit memory should be related to the overall strength of priming, while explicit memory scores relate more to lexical amplification.

## Method

This study comprised a baseline–training–posttesting design. Group (PWA vs. controls) was a between-subjects variable, while the training conditions (same verb vs. different verbs) and sessions (baseline, 1-day posttesting, and 1-week posttesting) were within-subject variables. PWA and age-matched controls were tested for general sentence production abilities before treatment commenced. During up to six training sessions, participants completed a structural priming task in which they were implicitly trained to produce passive and DO sentences.

### Participants

Twenty-four PWA and 16 neurologically healthy controls were included in this study. One further PWA and three control participants were recruited but were not enrolled in the study due to not meeting inclusion criteria or scheduling constraints. Of the 24 PWA, eight participants completed only one training sequence (dative training only), given their ceiling performance on passives at baseline, while the remaining 16 PWA (and all controls) completed the full training including passive and dative target sentences. All participants gave informed consent to take part and were compensated for their time. The study received approval from the Purdue University Institutional Review Board and is listed on ClinicalTrials.org (NCT05415501).

PWA and controls were matched for age (*M*_PWA_ = 59.2, *SD* = 11.4; *M*_Control_ = 63.4, *SD* = 11.1; *t* = −0.988, *p* > .05) and years in education (*M*_PWA_ = 16.3, *SD* = 1.79; *M*_Control_ = 16.7, *SD* = 1.35; *t* = −0.722, *p* > .05). All participants were native speakers of English. Participants passed a vision screening to ensure the absence of undue visual confounds and a hearing screening at 40 dB and 500–2000 Hz in at least one ear. We excluded participants who reported uncontrolled psychiatric/psychological conditions (e.g., depression, schizophrenia, attention-deficit/hyperactivity disorder), alcohol or substance abuse, or a history of acquired neurological conditions, such as dementia (other than stroke for PWA) that could affect daily communication and learning abilities. Controls further performed within normal limits for their age on the Cognitive Linguistic Quick Test Plus (CLQT+; Helm-Estabrooks, 2017).

PWA completed a set of clinical tests to determine eligibility. All participants included in the training followed the inclusion criteria as detailed in Lee, Van Boxtel, et al. (2025), and all PWA were at least 6 months postonset of their stroke (*M* = 81.3, *SD* = 66.7, range: 23–245 months). Formalized testing involved completing the Western Aphasia Battery–Revised (WAB-R; Kertesz, 2007), the Northwestern Assessment of Verbs and Sentences (NAVS; Cho-Reyes & Thompson, 2012), portions of the Comprehensive Aphasia Test (CAT; Swinburn et al., 2004), the Philadelphia Comprehension Battery (Saffran et al., 1988), and the Spoken Word–Picture Matching subtest of the Psycholinguistic Assessments of Language Processing in Aphasia (PALPA; Kay et al., 1996).

For a full overview of language test scores, see Table 1. PWA were eligible if they showed relatively intact comprehension of single words and simple sentences (> 70% on the PALPA Spoken Word–Picture Matching task, > 50% on the CAT Comprehension of Written Words subtest, > 6/10 WAB-R Auditory Comprehension subscore, > 80% on the NAVS Verb Comprehension Test, and > 40% on the NAVS Sentence Comprehension Test), and single-word naming of verbs and nouns (> 4/10 on the WAB-R Naming subscore and > 40% on the Verb Naming Test of the NAVS). Furthermore, PWA showed an ability to produce some grammatically simple (noun + verb) sentences (> 30% on the Argument Structure Production Test of the NAVS). Finally, the increasing word length and repeated trials subtests of the Apraxia Battery for Adults–Second Edition (Dabul, 2000) were administered, and those who exhibited severe apraxia of speech were excluded. PWA passed informal screening for significant attention and memory deficits using the Symbol Cancellation and Design Memory subtests of the CLQT+ (Helm-Estabrooks, 2017).

**Table 1.** Language testing scores for persons with aphasia, with means (μ) and standard deviations (σ^2^) included.

ID	WAB-R					PALPA	NAVS					CAT	PCB
	AQ	Fluency	AC	Naming	Repetition	SWP %	VCT	VNT	ASPT	SPPT	SCT	Comprehension of written words (%)	Total (%)
1	93.6	9	10	8.8	10	93	100	81.8	87.5	93.3	93.3	87	97
2	73.6	6	6.4	8.8	7.6	98	100	95.5	96.9	76.7	93.3	80	78
3	92.8	9	10	9.0	9.4	98	100	77.3	100	96.7	100	100	100
4	82.7	6	9.9	8.1	9.4	98	95.5	81.8	96.9	93.3	100	87	100
5	91.7	9	10	8.9	10	100	100	81.8	100	96.7	100	93	98
6	65.3	4	8.5	6.5	7.7	98	100	40.9	56.3	16.7	96.7	87	88
7	81.4	6	8.9	8.6	8.2	93	100	59.1	84.4	56.7	66.7	100	88
8	78.1	5	9.6	7.8	8.7	98	100	50.0	87.5	76.7	96.7	87	93
9	78.9	6	9.7	7.9	6.9	98	100	81.8	87.5	76.7	86.7	87	90
10	77.7	6	7.9	9.2	6.8	93	100	100	93.8	60.0	73.3	93	88
11	63.5	8	6.5	4.4	5.9	80	81.8	50.0	81.3	3.3	66.7	80	63
12	64.5	5	8.0	5.1	6.2	85	90.9	54.6	84.4	16.7	70.0	73	80
13	75.7	4	9.0	7.8	9.1	98	95.5	63.6	93.8	86.7	93.3	93	88
14	92.0	9	9.8	9.4	8.8	98	100	90.9	100	90.0	93.3	87	95
15	93.2	9	9.9	8.8	9.9	100	100	90.9	100	90.0	100	80	95
16	76.3	6	8.0	7.8	8.4	85	90.9	63.6	78.1	16.7	66.7	73	70
17	72.0	5	7.8	7.9	7.3	93	100	72.7	87.5	40.0	70.0	87	77
18	74.6	6	7.4	8.8	7.1	93	90.9	72.7	78.1	63.3	90.0	93	72
19	85.6	6	10	9.1	8.7	98	100	95.5	96.9	96.7	93.3	100	95
20	69.9	4	8.5	8.9	5.6	100	100	81.8	81.3	3.3	53.3	100	72
21	71.4	6	7.6	8.5	5.6	93	95.5	86.4	90.6	10.0	43.3	100	75
22	87.1	6	9.8	9.2	9.6	95	100	100	93.8	96.7	100	93	100
23	79.2	6	9.8	7.5	7.3	98	100	86.4	93.8	60.0	93.3	87	93
24	78.9	6	8.3	8.1	9.1	95	95.5	45.5	87.5	70.0	76.7	80	78
μ	79.2	6.3	8.8	8.1	8.1	94.9	97.3	75.2	89.1	62.0	84.0	88.6	86.4
σ^2^	9.2	1.6	1.2	1.2	1.4	5.2	4.6	17.8	9.9	33.6	16.4	8.2	11.0

*Note.* WAB-R = Western Aphasia Battery–Revised; PALPA = Psycholinguistic Assessments of Language Processing in Aphasia; NAVS = Northwestern Assessment of Verbs and Sentences; CAT = Comprehensive Aphasia Test; PCB = Philadelphia Comprehension Battery; AQ = Aphasia Quotient; AC = Auditory Comprehension; SWP = Spoken Word–Picture Matching Test; VCT = Verb Comprehension Test; VNT = Verb Naming Test; ASPT = Argument Structure Production Test; SPPT = Sentence Production Priming Test; SCT = Sentence Comprehension Test.

### Materials

The priming training targeted improved production of passive (e.g., “The cowboy is kicked by the angel”) and DO dative (e.g., “The magician is feeding the witch the pudding”) sentences. As these noncanonical structures are difficult for PWA and less frequently used by them (Cho & Thompson, 2010; Hartsuiker & Kolk, 1998; Lee, Man, et al., 2019), increased production of these sentence structures following structural priming training would indicate that structural priming successfully resulted in lasting effects in our participants.

**Figure 1.**
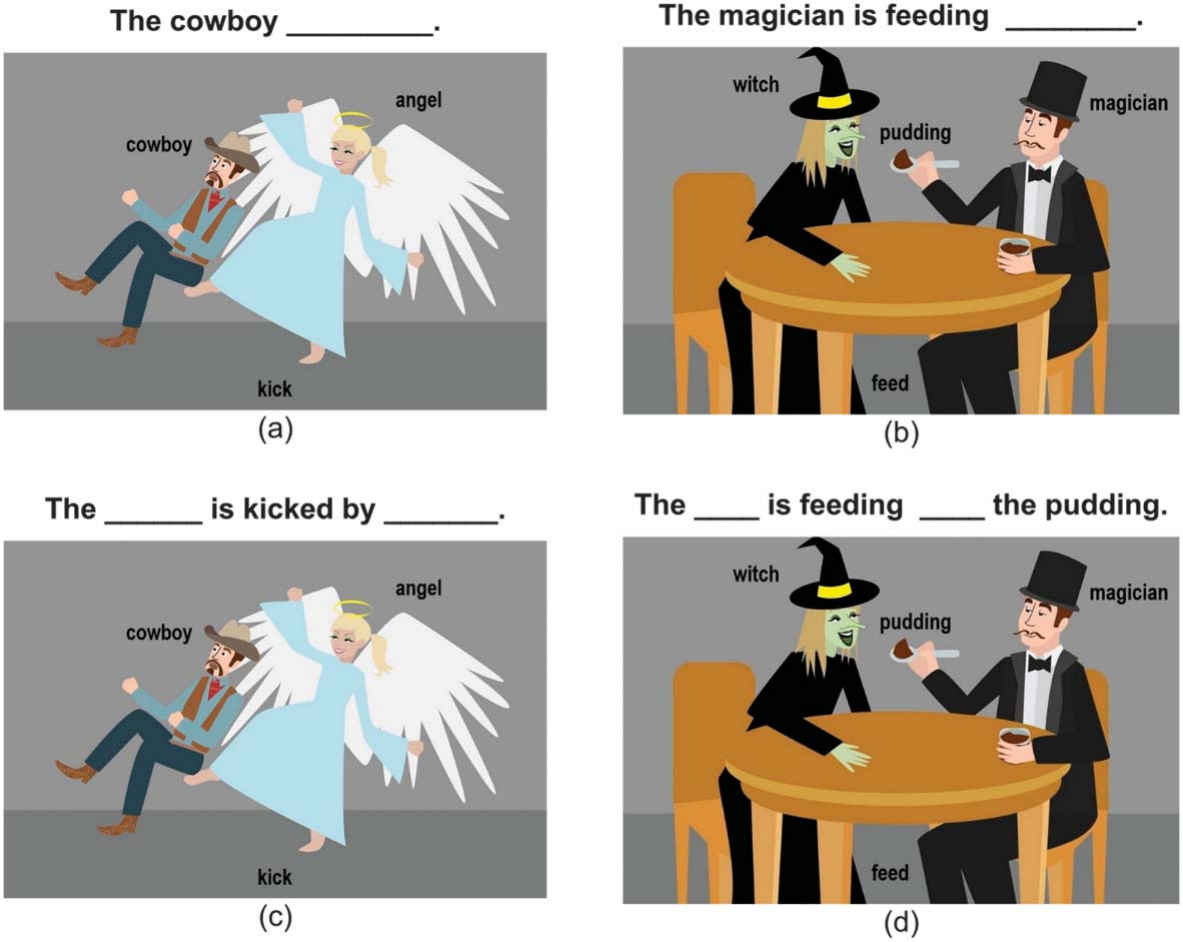
Example sentence production trials for passive (a) and DO (b) targets, and example sentence completion trials for passive (c) and DO (d) targets. DO = double-object.

Pictures were prepared for baseline, training, and posttesting sessions. These pictures included 10 transitive verbs (e.g., *pinch*) and 10 ditransitive verbs (e.g., *serve*), with each verb occurring in four repetitions involving different agent, theme, and goal (for datives) nouns. This created a total of 40 passive and 40 DO target pictures. See Figure 1 for examples of picture stimuli. Each verb–noun combination was unique, although nouns were repeated up to four times across all sentences. Phonological and semantic overlap between nouns used in each picture was avoided wherever possible, and the position of actors in the pictures was counterbalanced such that the agent appeared on the left side in 50% of pictures and on the right side in the other 50%. Target nouns and verbs were printed on the picture to ameliorate word retrieval deficits in the PWA group.

Half of the picture stimuli (but none of the pictures used to test generalization) were used as primes in the training blocks, while the other half were used as targets. Two primes were presented for each target, so each prime was presented twice in one training block: once as the first prime and once as the second prime. Finally, we prepared 40 filler sentences and pictures consisting of predicate adjective structures (e.g., “The purse is nice”), which appeared in all training sessions. Fillers were included to obscure the purpose of the training sessions and prevent participants from recognizing the repetition of passive and DO structures.

Lastly, we prepared a set of novel picture stimuli to test generalization to untrained items. These pictures were constructed in the same manner as baseline pictures (see above) but included verbs and nouns that were not presented in training sessions. Twenty such pictures were included in the sentence production task, and 10 were included in the sentence completion task.

#### Baseline and Posttesting

During baseline and all posttesting sessions, participants completed sentence production and sentence completion probe tasks. The sentence production probe included 60 total trials (30 passive and 30 DO), with each trial consisting of a picture and a corresponding sentence frame to ensure participants began their sentences with the intended actor (e.g., “The cowboy ______.” for the target “The cowboy is kicked by the angel.”) as shown in Figures 1a and 1b. The sentence production probe assessed independent production of the sentence predicate structure. The sentence completion probe included 40 trained (20 passive and 20 DO) and 20 untrained (10 passive and 10 DO) trials. Different from the sentence production task, the sentence completion task specifically tapped thematic role assignment between the two animate nouns (see Figures 1c and 1d). Thus, baseline and posttesting sessions tested sentence production abilities under these two different elicitation conditions.

#### Cognitive Testing

Three cognitive tests were built to gauge individual differences in implicit and explicit memory capacity. These tests were administered during baseline sessions. Implicit memory was assessed using a serial reaction time (SRT) task, which past research has shown is sensitive to implicit memory in PWA (Schuchard & Thompson, 2014), and a fragmented picture test (FPT; see Kessels et al., 2011). The SRT for this study involved four line drawings (of a shoe, knife, cake, and lamp) and audio recordings of each word spoken aloud. During each trial, the four drawings were presented on screen, and an audio recording of one word was played. The participants were tasked with pressing a button corresponding to the word they heard. An eight-trial sequence was developed (e.g., cake, knife, lamp, knife, shoe, cake, shoe, lamp) and repeated eight times in one block (64 trials per block), allowing participants to implicitly learn the sequence. After six blocks of this pattern (384 trials), a randomly sequenced block (block 7) was presented, followed by one block in which the learned sequence was resumed. Following Schuchard and Thompson (2014), our main SRT measure was calculated as the difference between mean response times in the random block (block 7) and the last patterned block (block 6), also known as the *rebound* effect (Kidd, 2012).

The FPT for this study included eight pictures presented in eight decreasing orders of fragmentation. The participants were told to name the object in the picture as soon as they recognized what the object was. This sequence of eight pictures was repeated three times in order, with the aim of participants recognizing the objects at earlier fragmentation stages during the second and third repetition. Additionally, a fourth follow-up repetition was administered approximately 15 min after the third repetition to gauge retention of the picture sequence. Our final FPT measure was the difference in average fragmentation stage between the first and the fourth repetition for each participant.

Explicit, verbal working memory was measured using the picture pointing span (PPS) task from the Temple Assessment of Language and Short-Term Memory in Aphasia (Martin et al., 2018). Nine line drawings of single-syllable objects (e.g., tree, book, ring, sun) were prepared, along with audio recordings of these words. The words were presented in increasing spans of two words (e.g., “apple, cat”), three words (“apple, cat, ring”), and four words (“apple, cat, ring, sun”). Participants' task was to click on the pictures matching the audio in the order they heard them. Four trials were presented for each span (4 × 2 words, 4 × 3 words, 4 × 4 words), and two practice trials preceded the main task. PPS scores were calculated as the number of correctly recalled words in the correct order.

#### Structural Priming Treatment Task

**Figure 2.**
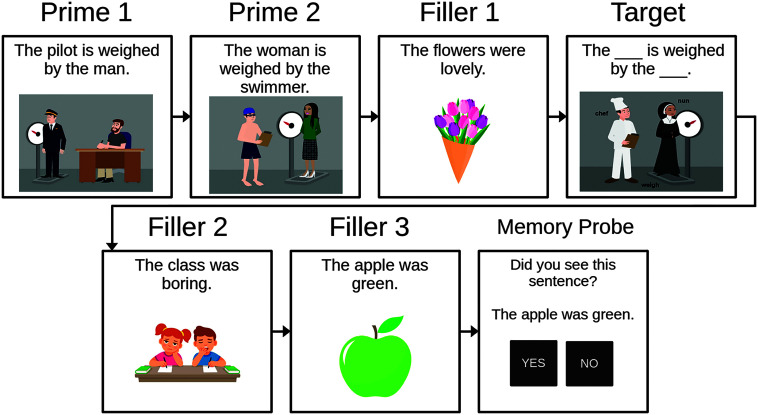
Schematic sequence of a passive same-verb prime training trial, comprising two primes, a filler, the target sentence completion, two further fillers, and a recognition probe.

See Figure 2 for a schematic overview of a training trial during the structural priming treatment. Training trials comprised seven different screens: two primes, one filler, one target, two additional fillers, and a recognition probe as shown in Figure 2. Prime screens consisted of pictures and the accompanying prime sentence, which participants heard and then read aloud. Recordings of the prime sentences were made by a female native speaker of English and were normalized at 70 dB. Then, the participants heard and read a filler sentence prior to completing the target sentence. Two further filler sentences were included to keep the nature of the priming task implicit and to minimize use of the same sentence structure throughout the training. Recognition probes were presented following each trial to encourage participants to attend to the task; these probes asked “Have you seen this sentence before?” The recognition probes included 10 filler sentences that the participants had read previously and 10 new intransitive sentences, thus requiring an affirmative response in half of all trials and a negative response in the other half.

Two priming conditions (same verb vs. different verb) were created for training sessions, for each structure separately. In the *same-verb priming* condition, both primes and the targets contained the same verb, thus allowing lexically mediated structural priming. In the different-verb priming condition, different verbs were used between primes and targets, such that the priming effect arose from the shared syntactic structure only. Each condition contained 20 trials and was repeated twice within a session. Thus, if a participant was trained in the same-verb condition for datives, they received three sessions of 40 same-verb dative trials each. All sentences across all structures and conditions are given in Supplemental Material S1 at https://osf.io/d9sxk/. For a schematic overview of the sequence of a training trial, see Figure 2. Training sessions further included a daily probe, which immediately followed the priming task, and consisted of 10 sentence production trials. These daily probes allowed us to measure improvements in independent sentence production as training was ongoing.

All controls and 16 of 24 PWA were randomly assigned to one of four lists with the priming condition and target structures crossed: (a) DO–same, passive–different; (b) DO–different, passive–same; (c) passive–same, DO–different; and (d) passive–different, DO–same. These lists reflected the order in which each structure was trained (DO first, passive second, or vice versa) and the training conditions in which each structure was trained. During baseline testing, the remaining eight PWA performed at ceiling for passive trials and were thus assigned to be trained on DO structures only. Four of these PWA received DO–same training, while the other four received DO–different training.

### Design and Procedure

**Figure 3.**
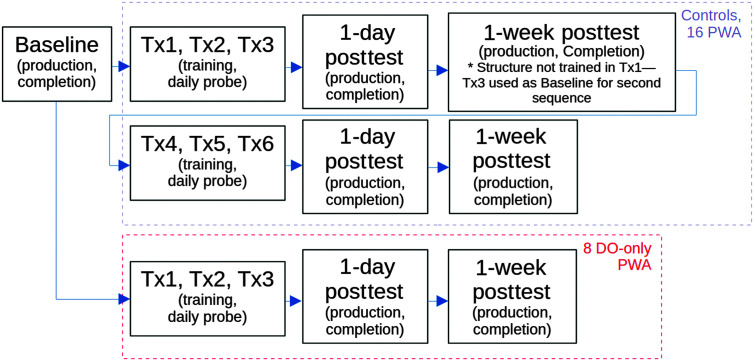
Schematic overview of the full study sequence. All controls and 16 PWA completed two sets of training (Tx), one in passive and one in dative (with the other alternating), while eight PWA who scored at ceiling on passives at baseline were trained on DOs only. PWA = persons with aphasia; DO = double-object.

The study was designed as a baseline–training–posttesting sequence, as shown in Figure 3. Participants completed initial baseline testing (cognitive testing, sentence production and completion), followed by up to six sessions of structural priming training (three sessions for each training condition), a 1-day posttest, and a 1-week posttest. Posttests exactly mirrored baseline testing, except that no further cognitive testing was conducted at posttests. Our main dependent variables of interest were, therefore, sentence production and completion accuracy at baseline compared to 1-day and 1-week posttesting, as well as accuracy on sentence production daily probes administered following training. Independent variables included priming training condition (same-verb, different-verb), group (PWA, control), and testing session (baseline, 1-day posttesting, and 1-week posttesting).

All tasks were coded and presented using Gorilla.sc (Anwyl-Irvine et al., 2020). Following baseline testing, participants first completed three training sessions for one structure (DO or passive) in one priming (same- vs. different-verb) condition, starting within 2 weeks from baseline and with no more than 4 days intervening between training sessions. Then, they completed a 1-day posttesting session (the day following the third training) and a 1-week posttesting session (exactly 1 week following 1-day posttesting). Then, within 2 weeks of this first delayed posttest, participants began training in the other structure (either DO or passive) under the other training condition, which again included three training sessions, 1-day posttesting session, and 1-week posttesting. For a schematic overview of this sequence, see Figure 3. Results from both sets of posttesting sessions were combined for 1 day and 1 week, respectively, as the two different target structures were used to avoid cross-condition priming effects (i.e., each structure was only ever trained in one condition for each participant). The eight DO-only PWA completed three training sessions each spaced no more than 4 days apart, followed by a posttesting session 1 day after Training Session 3, and a delayed posttesting session 1 week after the 1-day posttest. The other PWA (*n* = 16) and all age-matched controls (*n =* 16) were trained on both passives and datives. Analyses including only the 16 PWA who completed training in both structures did not differ from those including all 24 PWA. Therefore, we report data from all 24 PWA here.

Participants completed all sessions together with a trained researcher, either in person or via videoconferencing. Participants viewed the stimuli on an Apple Mac laptop with a 1920 × 1080 resolution if they participated in person, and on their own PCs or laptop if they took part virtually. The sequence of all tasks was controlled by experimenters: Experimenters advanced all screens, prompted for responses, and provided breaks. Experimenters were trained not to provide feedback on any aspect of baseline and posttesting tasks, except as general encouragement to participants to continue (e.g., “Great job, let's keep going.”) Experimenters did provide feedback on prime reading during the training blocks and prompted participants to retry prime reading if they made errors to ensure equal syntactic priming effects across participants.

#### Procedure for Baseline and Posttesting

Participants completed the sentence production task before the sentence completion task. The sentence production task aimed to assess the participant's production of sentence predicate structures while the sentence completion task assessed the ability to assign thematic roles to animate nouns, as mentioned above. During sentence production trials, participants were presented with the picture and sentence-initial frame and were instructed to build a sentence that corresponded to the picture they saw. They were asked to use the words printed on the picture and the sentence frame presented above the picture. The experimenter provided verbal prompts at each trial in the sentence production task: For passive trials, experimenters modeled the actors and the verb in the picture and emphasized the theme actor so participants would be encouraged to start their descriptions with the theme (e.g., “Here we have a cowboy, and angel, and the action is kick. What's happening with the cowboy here?” for the example in Figure 1a). For DO trials, only the actors and verb were modeled, and no further prompting was given (e.g., “Here we have a witch, a magician, some pudding, and the action is feed. What's happening in the picture?”). Following these prompts, the experimenter played a beep sound and the participant began their description. The sentence production task was split into six blocks of 10 trials each; blocks consisting of passive items alternated with blocks consisting of dative trials, and participants were encouraged to take a break after each block.

Sentence completion trials were split into four blocks (two passive and two DO) of 10 trials each; a passive block was always followed by a DO block, and vice versa. Completion trials were not preceded by any prompting, and participants were free to begin their descriptions as soon as they saw the picture and frame. For both sentence production and completion trials, picture screens timed out after 40 s, though participants were free to finish their responses after the picture had disappeared if they wished.

#### Procedure for Training Sessions

Training blocks began with a familiarization sequence, in which participants saw the nouns and verbs used in the training task written on the screen and heard them read by the experimenter. Participants were instructed to repeat the words after the experimenter. This sequence was followed before all training tasks to ameliorate word retrieval difficulties in PWA. Following familiarization, the first of four blocks of training trials (10 trials per block) was presented. Participants were told they would read and make sentences during priming training. They were instructed to repeat sentences they heard and, if they did not hear a sentence, to build a sentence for the picture they saw using the words printed on the picture. The experimenter clicked through the trials at the participant's pace. After the priming training concluded, daily probe trials were presented, which mirrored sentence production trials procedure-wise. Feedback given during training was minimal to emphasize the implicit nature of structural priming. Feedback was only given on oral reading accuracies of individual words in prime sentences, when needed. No feedback or instructions were provided on the syntactic structure of either prime reading or target sentence production.

### Data Transcription and Scoring

Participants' verbal responses in all tasks were audio-recorded through Gorilla.sc and transcribed verbatim, including repetitions, self-corrections, and filled pauses by trained researchers. For examples of transcription conventions, see numbers (1–4) below. When self-corrected attempts were made, the final attempted sentential response was scored. A sentential response was defined as the production of at least the subject noun and a lexical verb. Sentential responses, which had begun within the 40-s time limit but were finished outside this limit, were included in scoring and analysis. This 40-s boundary was marked with [//] when appropriate. Responses were considered “correctly produced target” if they included all the target nouns and the verb in either a passive (e.g., “The boxer is chased by the girl”) or a DO sentence structure (e.g., “The boy is giving the pirate a gift”). Although our dative sentence production task permitted either DO or PO structures, we took only DO responses as our preferred “correct” response and considered production of PO structures as a “nontarget” response, given that our priming training specifically targeted the DO structure. Phonological paraphasias, which did not affect the meaning of the target sentence, were accepted (e.g., “shelling” for “selling”) and marked “{pp}” in transcriptions, and synonyms (e.g., “guy” for “man”) were also accepted. Omissions of articles (“the/a(n)”) and auxiliary verbs were tolerated (e.g., “The cowboy kicked by the angel” or “The witch feeding the magician the pudding”), as were variations in verb tense (e.g., “was/is kicked by” or “feeds/is feeding/fed”). However, passive productions were scored correct only if the past participle marker –*ed* and the word “by” were present for both groups of participants.

1. Correct passive target, final sentential response scored: “(The nun was weighing the chef no the nun weighs the chef no the nun) the nun is weighed by the chef.”
2. Incorrect passive target, omission of passive morphology: “(The pirate was getting pat uh the pirate was being … ) the pirate was being pat by the monk.”
3. Correct DO target, including phonological paraphasia: “The referee is feeding the solier {pp} some yogurt.”
4. Incorrect DO target, argument structure error: “The (athlite athlite) athlete (is reading the spee) is reading to the mailman the speech.”

### Fidelity and Reliability

The study team established and maintained fidelity at each quarterly point of the study duration. Initial screenings and eligibility language testing sessions were administered or supervised by study personnel with master's level training in clinical aphasia assessment. Experienced clinicians (J.L., E.B.) reviewed language testing scores and resolved any inaccuracies to determine each participant's eligibility. All coders were required to demonstrate a minimum of 90% accuracy in transcription and coding in selected data sets prior to study involvement. For all training and probe sessions, a fidelity checklist was used to maintain the adherence to the training condition assignment, task sequence, the types of feedback, and instructions that each trainer provided. For the randomly selected 20% of the data, the study team demonstrated a 99.8% adherence rate.

Interrater reliability was continuously assessed throughout the data collection, and necessary training and adjustments were completed to ensure high reliability. Transcriptions and error types were double-checked by a second researcher who was not the initial coder. Discrepancies were resolved by discussions, and the study team was retrained for common errors throughout the study duration.

A random 20% of sessions were selected to compute interrater reliability, with two researchers independently scoring the data. Interrater reliability agreement was high at 96.29% for accuracy scoring (Cohen's *k* = .92) and 94.09% for error types (*k* = .87). Any residual errors were corrected prior to statistical analyses.

### Analysis

Our main analyses were threefold. First, we tested which training condition (same- vs. different-verb prime training) led to greater training gains in PWA and controls by examining accuracy on sentence production and completion trials at baseline, 1-day posttesting, and 1-week posttesting. Both target structures (passive and DO) were collapsed in this analysis: For participants who completed passive training first, their DO responses at delayed posttesting served as baseline for subsequent DO training, and vice versa. Production and completion accuracies were assessed by group (PWA vs. control), session (baseline vs. 1-day posttesting vs. 1-week posttesting), and training condition (same- vs. different-verb priming training).

Second, we assessed whether the participants would show sentence production improvements at each of the three priming training sessions and whether the results differed by training condition. For this analysis, we compared baseline data to sentence production accuracy from the daily probes administered at the end of each training session. Independent variables also included group, session (baseline vs. treatment session [Tx] 1, Tx2, and Tx3), and training condition.

Third, we analyzed whether priming effects transferred from trained to untrained stimuli. Both sentence production and sentence completion probes included picture stimuli that were presented in training sessions (exposed) and picture stimuli that participants did not see during training sessions (unexposed). This analysis included independent variables for group, training condition, and exposure (exposed vs. unexposed).

Finally, we related scores on the cognitive tests administered at baseline to training gains. All three scores (SRT, FPT, PPS) were centered and *z*-transformed prior to inclusion in any model and were included as interaction terms with group and session.

Analyses were run in R (R Core Team, 2023) and RStudio (RStudio Team, 2023); general data preparation and visualization were conducted in the “tidyverse” (Wickham, Averick, et al., 2019), “ggplot2” (Wickham, 2016), “data.table” (Dowle et al., 2019), and “readxl” (Wickham, Bryan, et al., 2019) packages. Binomial mixed-effects models were fitted using “lme4” (Bates et al., 2015) and evaluated using the “lmerTest” (Kuznetsova et al., 2017) and “emmeans” (Lenth et al., 2018) libraries. Variables were contrast coded as follows. For group, the reference level was control; for session, levels were coded such that baseline was compared against 1-day posttesting, and 1-day against 1-week posttesting; for training condition, the reference level was different-verb prime training; for exposure, the reference level was untrained. No models converged with random slopes of participant by item, but all models were constructed with maximum random intercepts for participant, item, and exposure (trained vs. untrained) whenever model convergence allowed.

## Results

This study comprised a baseline–training–posttesting design in which a structural priming training paradigm was used as a treatment for sentence-level abilities in PWA and age-matched controls. Participants (24 PWA, 16 controls) completed up to six sessions of structural priming training, following baseline sentence production and completion testing. These same tests were repeated following training to determine treatment gains. We evaluated (1) treatment effects from baseline to posttesting (1 day, 1 week) on independent sentence production and completion, which were our primary outcome measures; (2) gradual improvements on daily probe tasks taken immediately after training sessions; (3) generalization of treatment gains from items seen during training sessions to untrained items; and (4) effects of implicit and explicit memory scores on individual participants' treatment gains. One PWA could not complete cognitive tests due to technical issues; thus, for our analysis (4), we included 23 of 24 PWA. Two PWA could not complete daily probes in one training session and two training sessions, respectively, due to time constraints, resulting in a loss of 2.5% of daily probe data for the PWA group. All other PWA and controls completed all study tasks.

### Lasting Training Effects at 1-Day and 1-Week Posttesting

#### Sentence Production

**Figure 4.**
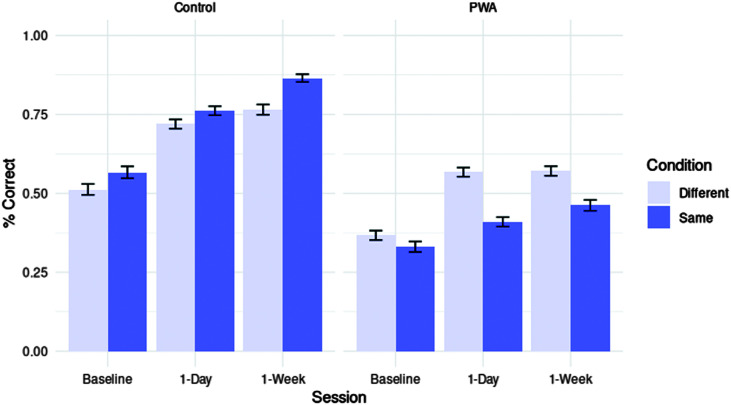
Accuracy on sentence production task by group (PWA vs. control), session (baseline vs. 1-day posttesting vs. 1-week posttesting), and training condition (same- vs. different-verb). PWA = persons with aphasia.

First, we assessed improvements in the sentence production task following training. A binomial mixed-effects model was fitted including fixed effects of group, session, and training condition and a random intercept for exposure. Adding an additional random intercept by participant prevented model convergence. For a full summary of this model, see Table 2, and for a visualization of effects, see Figure 4. Overall accuracy increased from baseline to 1-day posttesting (*z* = 11.256, *p* < .001, *OR* = 1.964) and from 1-day to 1-week posttesting (*z* = 6.674, *p* < .001, *OR* = 1.580). Considering training condition effects, overall accuracy was higher in the same-verb compared to the different-verb prime condition (*z* = 5.501, *p* < .001, *OR* = 1.450). The larger effect in the same-verb prime condition was also significant from 1-day to 1-week posttesting (*z* = 2.914, *p* < .01, *OR* = 1.356). However, PWA were less accurate in the same-verb condition compared to controls (*z* = −9.103, *p* < .001, *OR* = 0.457). Crucially, three-way interactions showed that PWA improved less than controls in the same-verb compared to the different-verb prime condition, both from baseline to 1-day posttesting (*z* = −3.402, *p* < .01, *OR* = 0.667) and from 1-day to 1-week posttesting (*z* = −2.575, *p* = .01, *OR* = 0.717).

**Table 2.** Binomial mixed-effects model summary predicting sentence production accuracy by group, session, and training condition.

Parameter	Est.	*SE*	*Z*	*p*	*OR*	Lower CI	Upper CI
Intercept	0.707	0.062				0.586	0.829
Group: PWA	−0.721	0.057	−12.640	**< .001**	0.486	−0.833	−0.610
Baseline to 1-day	0.675	0.060	11.256	**< .001**	1.964	0.557	0.792
1-day to 1-week	0.457	0.069	6.674	**< .001**	1.580	0.323	0.592
Training: Same	0.372	0.068	5.501	**< .001**	1.450	0.239	0.504
PWA × Baseline to 1-day	−0.126	0.079	−1.597	.110	0.882	−0.281	0.029
PWA × 1-day to 1-week	−0.175	0.085	−2.062	**.039**	0.839	−0.342	−0.009
PWA × Same	−0.783	0.086	−9.103	**< .001**	0.457	−0.952	−0.615
Baseline to 1-day × Same	0.153	0.090	1.700	.089	1.166	−0.023	0.330
1-day to 1-week × Same	0.305	0.105	2.914	**.004**	1.356	0.010	0.510
PWA × Baseline to 1-day × Same	−0.405	0.119	−3.402	**.001**	0.667	−0.639	−0.172
PWA × 1-day to 1-week × Same	−0.332	0.129	−2.575	**.010**	0.717	−0.585	−0.079

*Note.* Bold formatting indicates *p* < .05. *R*^2^_Marginal_ = .115, *R*^2^_Conditional_ = .116. This model contained a random intercept for exposure only (σ^2^ = 0.004, *SD* = 0.060). Est. = estimate; *SE* = standard error; *OR* = odds ratio; CI = 97.5% confidence interval; PWA = persons with aphasia.

#### Sentence Completion

**Figure 5.**
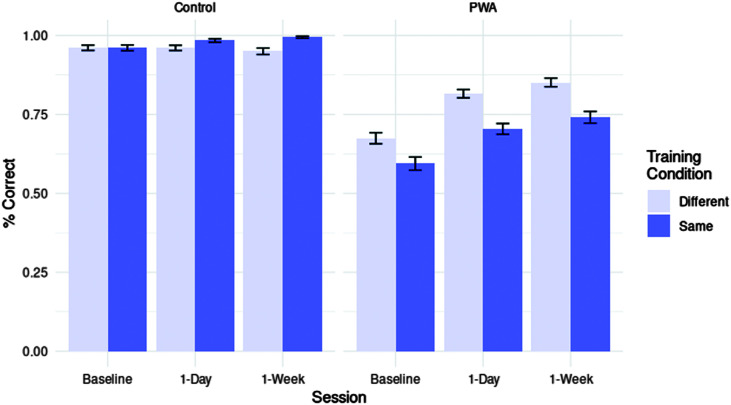
Accuracy on the sentence completion task by group (PWA vs. control), session (baseline vs. 1-day posttesting vs. 1-week posttesting), and training condition (same- vs. different-verb). PWA = persons with aphasia.

Analyses for the sentence completion task included PWA data only, due to controls scoring at ceiling on this task even at baseline (see Figure 5). Thus, no parameter for group was entered into the mixed-effects regression for sentence completion data. See Table 3 for a full overview of this model, which included random intercepts for participant and trial. PWA improved overall between baseline and 1-day posttesting (*z* = 10.347, *p* < .001, *OR* = 2.516) and from 1-day to 1-week posttesting (*z* = 6.622, *p* < .001, *OR* = 1.926). Overall, they were less accurate in the same-verb compared to the different-verb condition (*z* = −4.773, *p* < .001, *OR* = 0.649) and showed less improvement from baseline to 1-day posttesting in the same-verb compared to the different-verb condition, as well (*z* = −3.111, *p* < .01, *OR* = 0.677). There was further a marginal trend toward this effect from 1-day to 1-week posttesting (*z* = −1.673, *p* = .09, *OR* = 0.796).

**Table 3.** Binomial mixed-effects model summary predicting sentence completion accuracy by session and training condition.

Parameter	Est.	*SE*	*Z*	*p*	*OR*	Lower CI	Upper CI
Intercept	2.018	0.366				1.294	2.730
Baseline to 1-day	0.993	0.089	10.347	**< .001**	2.516	0.748	1.092
1-day to 1-week	0.655	0.099	6.622	**< .001**	1.926	0.461	0.849
Training: Same	−0.432	0.091	−4.773	**< .001**	0.649	−0.610	−0.255
Baseline to 1-day × Same	−0.390	0.125	−3.111	**.002**	0.677	−0.635	−0.144
1-day to 1-week × Same	−0.228	0.136	−1.673	.094	0.796	−0.496	0.039

*Note.* Bold formatting indicates *p* < .05. *R*^2^_Marginal_ = .035, *R*^2^_Conditional_ = .390. This model contained random intercepts for participant (σ^2^ = 2.891, *SD* = 1.700) and trial (σ^2^ = 0.261, *SD* = 0.511). Est. = estimate; *SE* = standard error; *OR* = odds ratio; CI = 97.5% confidence intervals.

### Daily Probe Results During Training Sessions

**Figure 6.**
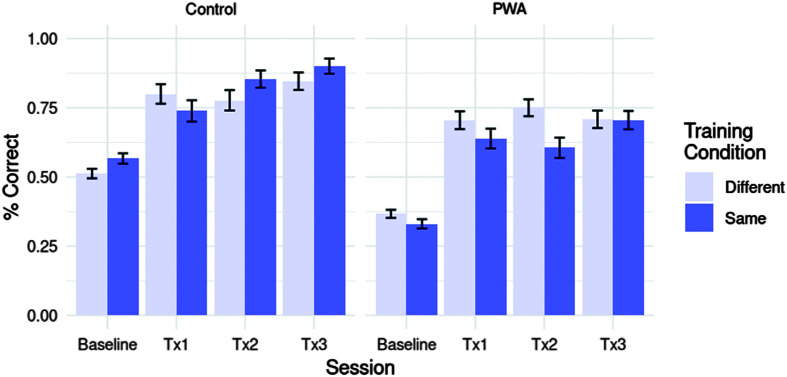
Accuracy on baseline sentence production and daily probes during training sessions by session (baseline vs. treatment session (Tx) 1 vs. Tx2 vs. Tx3), group (PWA vs. controls), and training condition (same- vs. different-verb). PWA = persons with aphasia.

Our next analysis, rather than assessing improvements following completion of training, examined gradual improvements while training was ongoing by comparing baseline sentence production data with performance on daily probes (administered at the end of each priming training session; see Figure 6).

The model for this analysis is summarized in Table 4. No random effects were fitted in this analysis, as any inclusion of random intercepts or slopes prevented model convergence. While PWA were less accurate than controls overall (*z* = −6.317, *p* < .001, *OR* = 0.551), accuracy across groups was higher at Tx1 than at baseline (*z* = 5.814, *p* < .001, *OR* = 3.805) and remained higher than baseline at Tx2 and Tx3 (*z*s > 5, *p*s < .001). Improvements between sessions were similar in both PWA and controls (*p*s > .05), indicating that both groups improved at similar rates between training sessions. Overall accuracy was higher in the same-verb compared to the different-verb prime condition (*z* = 2.112, *p* < .05, *OR* = 1.244). However, this effect was driven by the control group: PWA were, in fact, less accurate in the same-verb condition (*z* = −2.646, *p* < .01, *OR* = 0.686). Training condition differences were not reflected in by-session improvements, although a marginal trend existed for PWA to show less improvement from baseline to Tx2 in the same-verb compared to the different-verb prime condition (*z* = −1.929, *p* = .05, *OR* = 0.445).

**Table 4.** Binomial mixed-effects model summary predicting sentence production accuracy at baseline and posttraining probes by group, session, and training condition.

Parameter	Est.	*SE*	*Z*	*p*	*OR*	Lower CI	Upper CI
Intercept	0.050	0.069				−0.082	−0.186
Group: PWA	−0.595	0.094	−6.317	**< .001**	0.551	−0.780	−0.411
Tx1	1.336	0.230	5.813	**< .001**	3.805	0.901	1.805
Tx2	1.198	0.222	5.402	**< .001**	3.317	0.776	1.648
Tx3	1.655	0.253	6.548	**< .001**	5.231	1.181	2.177
Training: Same	0.218	0.103	2.112	**.035**	1.244	0.016	0.421
PWA × Tx1	0.080	0.285	0.281	.779	1.083	−0.488	0.630
PWA × Tx2	0.446	0.283	1.577	.115	1.562	−0.115	0.995
PWA × Tx2	−0.223	0.302	−0.740	.459	0.800	−0.833	0.355
PWA × Same	−0.377	0.143	−2.646	**.008**	0.686	−0.657	−0.098
Tx1 × Same	−0.567	0.314	−1.804	.071	0.568	−1.188	0.046
Tx2 × Same	0.299	0.342	0.875	.381	1.349	−0.364	0.980
Tx3 × Same	0.274	0.403	0.681	.496	1.316	−0.504	1.087
PWA × Tx1 × Same	0.425	0.395	1.075	.282	1.530	−0.348	1.203
PWA × Tx2 × Same	−0.809	0.420	−1.929	.054	0.445	−1.641	0.008
PWA × Tx3 × Same	−0.129	0.470	−0.274	.784	0.879	−1.066	0.782

*Note.* Bold formatting indicates *p* < .05. *R*^2^ = .116. This model did not converge with any random effects. Tx = treatment session; Est. = estimate; *SE* = standard error; *OR* = odds ratio; CI = 97.5% confidence intervals; PWA = persons with aphasia.

### Generalization to Untrained Items

Both the sentence production and completion tasks included novel stimuli that were not exposed during training sessions. To evaluate whether structural priming was effective in generating improvements to the sentence production systems of PWA in general, rather than to item-specific learning, we assessed whether there were differences between exposed (trained) and unexposed (not presented during training sessions) stimuli at posttesting compared to baseline. This was assessed using (a) model comparisons between full models predicting accuracy, including a three-way interaction of group, session, and exposure on the one hand, and a null model containing a two-way interaction of group and session as well as a main effect of exposure, and (2) evaluations of the model output for the full models (see Table 5).

**Table 5.** Binomial mixed-effects model summaries predicting sentence production (both groups) and sentence completion accuracy (persons with aphasia [PWA] only) by group (production only), session, and exposure during training.

Task	Parameter	Est.	*SE*	*Z*	*p*	*OR*	Lower CI	Upper CI
Sentence production	Intercept	0.795	0.162				0.478	1.113
	Group: PWA	−0.681	0.184	−3.698	**< .001**	0.506	−1.042	−0.320
	Baseline to 1-day	0.856	0.058	14.667	**< .001**	2.353	0.741	−0.970
	1-day to 1-week	0.655	0.066	9.963	**< .001**	1.925	0.526	0.784
	Exposure: Unexposed	−0.047	0.074	−0.614	.521	0.954	−0.192	0.097
	PWA × Baseline to 1-day	−0.324	0.077	−4.223	**< .001**	0.723	−0.475	−0.174
	PWA × 1-day to 1-week	−0.378	0.081	−4.643	**< .001**	0.685	0.538	−0.219
	PWA × Unexposed	−0.145	0.094	−1.537	.124	0.865	−0.329	0.040
	Baseline to 1-day × Unexposed	−0.002	0.099	−0.022	.983	0.998	−0.197	0.193
	1-day to 1-week × Unexposed	0.022	0.113	0.192	.848	1.022	−0.199	0.243
	PWA × Baseline to 1-day × Unexposed	−0.181	0.101	−1.386	.166	0.834	−0.438	0.075
	PWA × 1-day to 1-week × Unexposed	−0.024	0.140	−0.173	.863	0.976	−0.299	0.250
Sentence completion	Intercept	1.920	0.378				1.180	2.660
	Baseline to 1-day	0.811	0.087	9.333	**< .001**	2.250	0.610	0.981
	1-day to 1-week	0.638	0.904	6.759	**< .001**	1.892	0.453	0.823
	Exposure: Unexposed	−0.235	0.182	−1.295	.195	0.790	−0.592	0.121
	Baseline to 1-day × Unexposed	−0.178	0.121	−1.473	.141	0.837	−0.456	0.059
	1-day to 1-week × Unexposed	−0.207	0.133	−1.563	.118	0.813	−0.467	0.053

*Note. *Bold formatting indicates *p* < .05. *R*^2^_Production, Marginal_ = .072, *R*^2^_Production, Conditional_ = .184; the sentence production model included a random intercept for participant (σ^2^ = 0.556, *SD* = 0.746). *R*^2^_Completion, Marginal_ = .032, *R*^2^_Completion, Conditional_ = .389; the sentence completion model included random intercepts for participant (σ^2^ = 2.938, *SD* = 1.714) and trial (σ^2^ = 0.262, *SD* = 0.512). Est. = estimate; *SE* = standard error; *OR* = odds ratio; CI = 97.5% confidence intervals.

For the sentence production data, the full model including the exposure interaction fitted the data no better than the null model, χ^2^(5) = 9.55, *p* > .05, BF_Exposure_ = 0.060, confirming that models containing exposure did not explain greater variance than models that did not. Assessing model output further showed that exposure did not affect levels of accuracy across groups, nor in each group separately, nor in any particular testing session (all *p*s > .05), which confirms significant generalization to untrained stimuli. Similar results were obtained for sentence completion, although given ceiling performance in controls, only PWA data were modeled. The full model fit the data no better than the null model, χ^2^(2) = 2.934, *p* = .231, BF_Exposure_ = 0.331. Similar findings resulted from the full model output (lower panel, Table 5): Exposure did not affect accuracy overall and did not interact with session (all *p*s > .05).

### Cognitive Testing Scores

Finally, we assessed whether performance on the SRT, FPT, and PPS tasks underpinned sentence production improvements through structural priming training. This analysis was limited to sentence production and did not include sentence completion data as controls performed at ceiling on the completion task. See Table 6 for a full overview of models (one for each cognitive test) evaluating sentence production accuracy and cognitive testing scores.

**Table 6.** Binomial mixed-effects model summaries predicting sentence production by cognitive testing scores, group, and session.

Test	Parameter	Est.	*SE*	*Z*	*p*	*OR*	Lower CI	Upper CI
SRT	Intercept	0.909	0.034				0.843	0.976
	Group: PWA	−1.010	0.043	−23.097	**< .001**	0.364	−1.096	−0.925
	Baseline to 1-day	0.751	0.046	16.347	**< .001**	2.118	0.661	0.841
	1-day to 1-week	0.603	0.053	11.461	**< .001**	1.826	0.500	0.706
	SRT scores	−0.119	0.075	−1.595	**.111**	0.888	−0.267	0.026
	PWA × Baseline to 1-day	−0.292	0.061	−4.799	**< .001**	0.747	−0.411	−0.172
	PWA × 1-day to 1-week	−0.306	0.065	−4.681	**< .001**	0.736	−0.435	−0.179
	PWA × SRT	0.376	0.078	4.825	**< .001**	1.456	0.225	0.530
	Baseline to 1-day × SRT	−0.043	0.095	–0.450	.653	0.958	−0.230	0.143
	1-day to 1-week × SRT	0.026	0.120	0.217	.828	1.026	−0.213	0.258
	PWA × Baseline to 1-day × SRT	0.099	0.100	0.988	.323	1.104	−0.097	0.297
	PWA × 1-day to 1-week × SRT	0.025	0.124	0.200	.842	1.025	−0.215	0.272
FPT	Intercept	0.831	0.035				0.762	0.901
	Group: PWA	−0.939	0.045	−20.816	**< .001**	0.391	−1.027	−0.851
	Baseline to 1-day	0.737	0.047	15.524	**< .001**	2.089	0.644	0.830
	1-day to 1-week	0.569	0.055	10.394	**< .001**	1.767	0.463	0.678
	FPT scores	0.352	0.052	6.806	**< .001**	1.422	0.252	0.455
	PWA × Baseline to 1-day	−0.199	0.063	−3.179	**.001**	0.819	−0.322	−0.076
	PWA × 1-day to 1-week	−0.226	0.068	−3.331	**.001**	0.798	−0.359	−0.094
	PWA × FPT	−0.174	0.058	−3.002	**.003**	0.840	−0.288	−0.061
	Baseline to 1-day × FPT	0.141	0.069	2.058	**.040**	1.152	0.007	0.276
	1-day to 1-week × FPT	0.042	0.086	0.488	.626	1.043	−0.124	0.213
	PWA × Baseline to 1-day × FPT	0.021	0.078	0.275	.783	1.022	−0.132	0.174
	PWA × 1-day to 1-week × FPT	−0.009	0.093	−0.092	.926	0.991	−0.194	0.172
PPS	Intercept	0.560	0.053				0.457	0.664
	Group: PWA	−0.562	0.061	−9.160	**< .001**	0.570	−0.682	−0.442
	Baseline to 1-day	0.273	0.073	3.740	**< .001**	1.314	0.130	0.416
	1-day to 1-week	0.180	0.082	2.191	**.028**	1.198	0.020	0.343
	PPS scores	0.511	0.063	8.090	**< .001**	1.667	0.387	0.634
	PWA × Baseline to 1-day	0.138	0.086	1.608	.108	1.148	−0.030	0.307
	PWA × 1-day to 1-week	0.034	0.094	0.361	.718	1.034	−0.151	0.217
	PWA × PPS	−0.270	0.070	−3.867	**< .001**	0.764	−0.406	−0.133
	B to 1-day × PPS	0.709	0.086	8.248	**< .001**	2.032	0.541	0.878
	1-day to 1-week × PPS	0.609	0.101	6.013	**< .001**	1.839	0.410	0.808
	PWA × Baseline to 1-day × PPS	−0.802	0.096	−8.340	**< .001**	0.448	−0.991	−0.614
	PWA × 1-day to 1-week × PPS	−0.752	0.110	−6.859	**< .001**	0.472	−0.967	−0.537

*Note.* Bold formatting indicates *p* < .05. All cognitive task models were fit without random effects due to model convergence issues. *R*^2^_SRT_ = .136; *R*^2^_FPT_ = .142, *R*^2^_PPS_ = .124. Est. = estimate; *SE* = standard error; *OR* = odds ratio; CI = 97.5% confidence intervals; SRT = serial reaction time; PWA = persons with aphasia; FPT = fragmented picture test; PPS = picture pointing span.

Sentence production improvements between baseline and 1-day posttesting and between 1-day and 1-week posttesting were maintained across groups in all models (*p*s < .001). PWA were more facilitated by high SRT scores than controls overall (*z* = 4.825, *p* < .001, *OR* = 1.456), but session-by-session improvements were not affected by SRT scores (*p*s > .05). High FPT scores were associated with higher accuracy overall (*z* = 6.806, *p* < .001, *OR* = 1.422) and with greater accuracy improvement from baseline to 1-day posttesting (*z* = 2.058, *p* < .05, *OR* = 1.152). PWA were less facilitated by FPT scores overall than controls (*z* = −3.002, *p* < .01, *OR* = 0.391), but a three-way Group × Session × FPT interaction was not significant (*p*s > .05). Analyses of PPS scores yielded somewhat different findings: While high PPS scores were associated with higher accuracy overall (*z* = 8.090, *p* < .001, *OR* = 1.667) as well as between baseline and 1-day posttesting, and 1-day and 1-week posttesting (*p*s < .001), PWA were less facilitated by PPS scores than controls (*z* = −3.867, *p* < .001, *OR* = 0.764). Crucially, a three-way Group × Session × PPS interaction further confirmed that PWA did not see as much PPS-facilitated improvement between baseline and 1-day posttesting (*z* = −8.340, *p* < .001, *OR* = 0.448) or between 1-day and 1-week posttesting (*z* = −6.859, *p* < .001, *OR* = 0.472).

### Individual Variability

**Figure 7.**
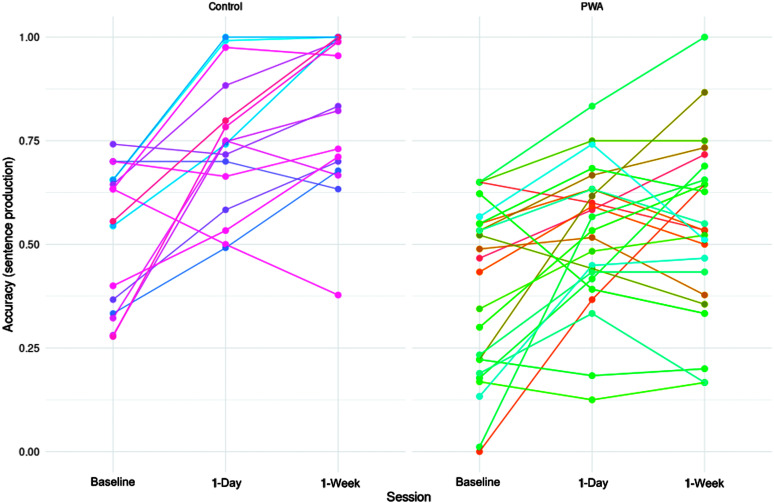
Accuracy on the sentence production task by participant, group, and session.

**Figure 8.**
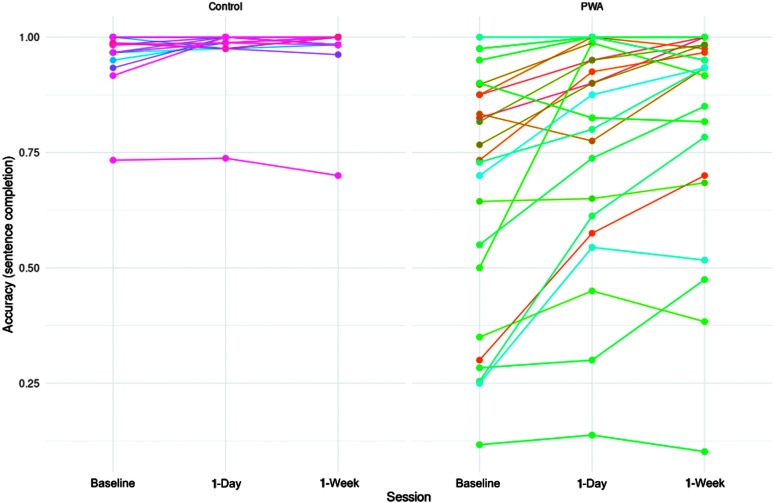
Accuracy on the sentence completion task by participant, group, and session.

Finally, we informally gauged interindividual variability within the sentence production and sentence completion tasks. Figures 7 and 8 display performance on the sentence production and completion tasks, respectively, arranged by participant and group. On the sentence production task, all but five PWA improved from baseline to 1-day posttesting, while all but three controls did. Maintenance of treatment gains to 1-week posttesting were more variable, although group-level analyses reported earlier confirm this maintenance was reliable (see the Sentence Production section). Results from the sentence completion task were similar (although note that control performance was at ceiling on this task). A majority of the PWA showed a trend for increased accuracies at 1-day posttesting compared to baseline, although the degrees of increases varied across individuals. Among those who showed the treatment effect, a majority of them retained the improvements at 1-week posttesting.

## Discussion

This study conducted a structural priming sentence production training procedure for PWA who presented with sentence production deficits. The study aimed to further validate structural priming as a viable treatment for aphasia, following Lee, Van Boxtel, et al. (2025), and to specify essential treatment components by including a verb overlap manipulation during training sessions. Participants completed either three or six priming training sessions during which they orally read passive and DO prime sentences and then completed sentences based on target pictures. Before training, baseline measures of participants' sentence production and completion accuracy were obtained; these measures were repeated 1 day after training ceased and again 1 week following the 1-day posttest. We hypothesized that structural priming training would lead to increased sentence production accuracy from pretraining to posttraining but made no specific hypotheses about the impact of verb overlap.

First, both PWA and age-matched controls showed significant sentence production accuracy gains from baseline to 1-day posttesting and maintenance of those gains at 1-week posttesting. This reinforces existing evidence suggesting structural priming is effective in creating long-lasting improvements in sentence production abilities of PWA (Cho-Reyes et al., 2016; Lee & Man, 2017; Lee, Van Boxtel, et al., 2025; Rainey et al., 2024; Saffran & Martin, 1997). Importantly, treatment gains were observed in PWA in both an independent sentence production task and a more thematic role-focused sentence completion task, in which participants filled in actor slots with available nouns. This further suggests that the act of “mapping” thematic roles to grammatical elements is specifically facilitated by priming. Improvements from one training session to the next were also assessed using our daily probe data, which showed cumulative treatment gains in PWA across sessions, further supporting the effectiveness of priming as a gradual facilitator of sentence production abilities. Priming-induced gains generalized to untrained items across groups and training conditions and were maintained from 1-day to 1-week posttesting without effects of exposure during training sessions. Although maintenance to 1-week posttraining does not reflect a retention of treatment gains to the very long term, past studies of structural priming treatments in aphasia are promising in this regard, with, for instance, Lee and Man (2017) showing reliable maintenance at 1-month posttraining. Future studies should continue to evaluate whether priming-induced gains are maintained. Our findings nevertheless constitute a substantial advance in sentence-level aphasia treatments, which have thus far mainly utilized repeated practice of scripted speech (e.g., script training; Goldberg et al., 2012) and explicit metacognitive training of grammatical rules (e.g., Rochon et al., 2005; Thompson et al., 2003), and suggests that structural priming lead to generalized improvements to sentence production abilities in PWA .

While both PWA and controls showed significant priming-induced gains, our findings nevertheless suggest these effects were not predominantly the result of general, task-specific learning: Conditional differences between same-verb and different-verb priming confirm that structural priming was, in fact, the mechanism of action underlying our treatment effects. While it cannot be ruled out that participants began to understand the priming task as they completed more sessions, their syntactic encoding systems were still implicitly facilitated by our priming conditions. In general, results from our lexical overlap manipulation were quite interesting, with controls and PWA showing different training condition effects. While controls showed greater improvements in the same-verb condition, the PWA participants included in this study showed the opposite effect. In healthy speakers, this lexical “boost” is thought to rely on the concurrent activation of syntactic structures and associated lexical information (e.g., Pickering & Branigan, 1998) and therefore relies on both lexical and syntactic encoding and processing. Our findings from the control group support the observation that, in healthy older adults, lexical boost effects manifest, in line with recent observations (e.g., Hardy et al., 2017, Lee et al., 2024, in production; Van Boxtel & Lawyer, 2023a, 2023b, in comprehension), and these boost effects contribute to longer term treatment effects. This may be because the connections between lexical and syntactic representations remain intact or accessible in healthy older adulthood. Another (nonmutually exclusive) possibility derives from observation in some studies, highlighting how abstract priming and the lexical boost may be underpinned by distinct memory components: Specifically, while the lexical boost is thought to be related to short-term, explicit memory, abstract priming may be subserved by implicit memory abilities (e.g., Chang et al., 2012; Hartsuiker et al., 2008; Tooley, 2020; Traxler et al., 2014). Our cognitive testing findings lend some support to this account: Controls, who showed a significant lexical boost, were further facilitated by high scores on our explicit memory measure, the PPS test. High scores on this test indicate larger explicit memory, specifically verbal working memory, spans. However, PWA, who showed no lexical boost effects, were not. This suggests that in controls, longer term treatment effects may have an explicit memory component, an effect that is reduced in PWA.

Indeed, PWA showed the opposite pattern to controls, both in sentence production and sentence completion measures as well as during priming training sessions. Across tasks and sessions, PWA showed greater treatment gains when verbs did *not* match between primes and targets compared to when verbs *did* match. Unlike the literature on healthy speakers, which has reported robust lexical boost effects when verbs in primes and targets overlap (Hartsuiker et al., 2008; Van Boxtel & Lawyer, 2023a, 2023b), past studies have painted an unclear image of lexical boost effects in PWA: Some have found reliable facilitation as a result of verb overlap (e.g., Lee et al., 2023; Yan et al., 2018), while others find null effects in PWA (e.g., Man et al., 2019; Van Boxtel et al., 2023). One reason why verb overlap here did not lead to larger treatment effects is that PWA may not have shown a basic lexical boost effect at all. Curiously, the results here go even further, showing *inverse* lexical boost effects, in which verb overlap leads to *smaller* treatment gains in PWA.

One might think that our research design was not optimized for capturing lexical boost effects due to the baseline–training–posttesting setup we used. If lexical boost effects are as short-lived as much of the literature suggests (e.g., Hartsuiker et al., 2008), then these effects may not have lasted long enough for them to facilitate sentence production 1 day and 1 week after training ceased. That is, only abstract structural priming could have transferred to posttesting sessions. We do not discount this possibility; however, an informal analysis of target production accuracy during training sessions (when the temporal lag between prime exposure and target production was minimal) also showed greater priming effects for PWA in the different-verb condition, with the opposite pattern for controls. The inverse lexical boost effect we report therefore seems consistent even when taking temporal lag into account.

We also offer a few possible explanations outside of our research design for why the same-verb prime training condition did not yield greater training gains in PWA. First, impairments in the lexical activation system in PWA may prevent their linguistic encoding systems from benefiting from lingering activation of lexical information. Lexical processing impairments are widespread in PWA and may manifest as slowed activation of lexical representations (Ferrill et al., 2012; Mack et al., 2013; Prather et al., 1992); this—if the lexical boost relies on the concurrent activation of lexical and syntactic information—may be a plausible explanation for the absence of lexical boost effects in PWA. Relatedly, lexical deficits in aphasia may include difficulties integrating lexical information in context (e.g., Choy & Thompson, 2010; Swaab et al., 1997), which further implicates the lexical–syntactic interactions crucial to the lexical boost as a potential locus for deficits in PWA. Again, without a basic lexical boost effect on priming, no boost effect is expected on treatment measures as well.

Another possible explanation is rooted in the memory systems subserving priming, as discussed above. Given that at least on some accounts, the lexical boost is thought to rely on different, more explicit memory systems than nonlexical (abstract) priming alone (Chang et al., 2012; Hartsuiker et al., 2008; Reitter et al., 2011), PWA in this sample may have experienced competition and interference from concurrently active memory systems. If the memory systems underlying abstract priming and the lexical boost are indeed different and competitive, priming-induced treatment gains may have been dampened by this competition when both systems are active concurrently, compared to when only implicit, abstract priming is active (Ferreira & Bock, 2006; Heyselaar et al., 2017). This possibility is supported by evidence suggesting the lexical boost decays rapidly following prime sentence reading or listening, while abstract, nonlexical priming persists much longer (Branigan & McLean, 2016; Chang et al., 2006; Hartsuiker et al., 2008; Man et al., 2019), and by research showing reduced short-term verbal memory in PWA compared to age-matched controls (e.g., Martin et al., 2018; Mayer & Murray, 2012; Salis et al., 2017). Indeed, our inclusion of memory analyses sheds some light on this possibility: While both controls and PWA were facilitated by high implicit memory metrics (represented by FPT scores), only controls showed greater treatment gains with higher explicit memory scores (PPS). This may be related to training condition effects, especially if PWA were unable to balance the competing demands of implicit and explicit memory or could not leverage their explicit memory systems to facilitate lexically mediated priming.

Lastly, studies on learning mechanisms suggest acquisition effects can be enlarged by creating “desirable difficulty”—that is, more learning occurs when the material to be learned is associated with varying learning conditions, including environmental settings, task formats, time between learning sessions, learned exemplars, and the number of opportunities for reactivation of learned material (E. L. Bjork & Bjork, 2011; Bjork, 2017; Nelson & Eliasz, 2023). In the current study, the different-verb priming condition would have been associated with greater “desirable” difficulty, as three different verbs were presented in each prime–prime–target trial sequence during training. Conversely, the same-verb condition would have been associated with less difficulty, as all three verbs were identical. Following this account, the “easier” same-verb condition would not have elicited as much priming-induced improvement than the more difficult different-verb condition. Related evidence from a child population is presented by Savage et al. (2006), who similarly uncovered greater and longer lasting priming effects when verbs varied between primes and targets compared to when verbs were identical. They attribute this inverse lexical boost effect to processes of abstraction: By introducing greater variation in the lexical–syntactic material to which speakers are exposed, the process of generalizing learned examples to wider contexts and set grammatical rules is improved (Savage et al., 2006). It could be that grammatical learning in PWA was similarly facilitated by greater variation in lexical–syntax representations in the experienced input in our study. This suggestion is somewhat tentative, given that our controls did not show greater learning in the different-verb condition and that it remains unclear the extent to which implicit nature of priming is susceptible to such learning strategies. Future research should explore how different “difficulties” associated with priming paradigms affect the magnitude of acquired gains.

While verb overlap may therefore not constitute a facilitating component for a structural priming treatment (and indeed, treatments may benefit from an absence of verb overlap), several other possible components and mechanisms remain to be explored. For example, longer term maintenance of structural priming treatment gains were not explored beyond 1 week following training cessation in this study. While reports from single-subject–based treatment studies suggest priming-induced gains are long lasting up to 2 months (Lee & Man, 2017; Rainey et al., 2024), a comprehensive, quantitative evaluation of the longevity of training effects is still warranted. Similarly, while this study did achieve full generalization of treatment gains from trained to untrained items, we did not investigate whether these gains generalized to other modalities. Cross-modality treatment generalization is not consistently achieved by current sentence-level aphasia treatments (see Adelt et al., 2018, for a review), and past research suggests structural priming is effective across modalities (e.g., Litcofsky & Hell, 2019; Mahowald et al., 2016, for controls; see also Keen & Lee, 2024, for aphasia). Determining whether priming-induced improvements generalize to other modalities in PWA will therefore be an important topic for future research.

This study is not without limitations, which warrant further research. First, our PWA sample included only individuals with moderate-to-mild aphasia, as we chose to target noncanonical sentences, as measuring PWA's ability to produce noncanonical sentences is a well-established empirical method to assess syntactic abilities. However, as mentioned in our introduction, structural priming effects are not restricted to complex sentences and have been manifested in various simpler structures as well (e.g., see Mahowald et al., 2016, for meta-analysis; also see Rainey et al., 2024, for structural priming treatment for transitive active sentences in PWA). Therefore, future research should expand clinical utility of structural priming treatment in more severe PWA. Second, while our test of generalization from trained to untrained items provides solid evidence that treatment gains were not item-specific, more extensive explorations of generalization to other communication tasks and contexts were not included. This is a prime avenue for future research into the applicability and effects of structural priming-based treatments: Whether generalization from treatment affects domains such as narrative sentence production, fluency, or interpersonal conversational skills remains to be explored.

## Conclusions

In short, this study makes a strong case for the further development of structural priming paradigms as treatment components for sentence production deficits in aphasia. Treatment effects were widespread, occurred across sentence elicitation tasks, and persisted until a week after training ceased, replicating Lee, Van Boxtel, et al. (2025). Acquisition was stronger in PWA when training sessions included no verb overlap between prime and target sentences, while controls benefited more from the same-verb prime training. Our results therefore do not support the use of verb overlap as an effective facilitator for structural priming in aphasia and indeed suggest that stronger treatment effects result from different-verb training. Future studies should continue to validate structural priming as a treatment for sentence production impairments and should investigate further treatment outcomes, such as generalizability to untrained structures and modalities, and longevity and long-term maintenance of priming-induced treatment gains.

## Author Contributions

**Willem S. van Boxtel:** Conceptualization, Methodology, Investigation, Data curation, Formal analysis, Writing – original draft, Visualization, Project administration. **Katelin M. Rainey:** Investigation, Data curation, Project administration. **Victor Ferreira:** Conceptualization, Funding acquisition, Writing – review & editing. **Nadine Martin:** Conceptualization, Funding acquisition, Writing – review & editing. **Emily Bauman:** Investigation, Data curation, Project administration. **Jiyeon Lee:** Supervision, Conceptualization, Methodology, Investigation, Funding acquisition, Project administration, Writing – review & editing.

## Data Availability Statement

Supplemental materials for this study, including full study data, are available on the Open Science Framework at https://osf.io/d9sxk/.
